# Tunable odd-frequency triplet pairing states and skyrmion modes in chiral p-wave superconductor

**DOI:** 10.1038/s41598-017-10152-0

**Published:** 2017-08-29

**Authors:** Yu-Feng Lou, Lin Wen, Guo-Qiao Zha, Shi-Ping Zhou

**Affiliations:** 1 Department of Physics, Shanghai University, 99 Shangda Road, Shanghai, 200444 China; 2 Shanghai Key Laboratory of High-Temperature Superconductor, Shanghai, 200444 China

## Abstract

Bogliubov-de Gennes equations are solved self-consistently to investigate the properties of bound states in chiral p-wave superconductive disks. It shows that either an s-wave or the mixed d- and s-wave state with odd-frequency and spin-triplet symmetry is induced at the vortex core, depending both on the chirality of the pairing states and on the vortex topology. It is also found that the odd-frequency triplet even parity (OTE) bound state can be manipulated with a local non-magnetic potential. Interestingly, with an appropriate potential amplitude, the zero-energy OTE bound state can be stabilized at a distance from the vortex core and from the local potential. Possible existences of the Majorana fermion modes are expected if the particle-hole symmetry property is applied to the zero-energy OTE bound state. Moreover, skyrmion modes with an integer topological charge have been found to exist.

## Introduction

The layered ruthenate superconductor *Sr*_2_ *RuO*_4_ is the first perovskite compound showing superconductivity without cupper-oxide planes^1^. Experimental and theoretical investigations suggest that its superconducting pairing state is most-likely a spin-triplet chiral p-wave state with the *p*_*x*_ ± *ip*_*y*_ symmetry^2–5^. In particular, Nelson *et al*.^6^ performed the measurements to confirm an odd-parity and spin-triplet Cooper pairing symmetry in *Sr*_2_ *RuO*_4_ superconductor. The *p*_*x*_ ± *ip*_*y*_ state that has an intrinsic orbital angular momentum *L* = ±1 and breaks down the time-reversal symmetry has shown promising new physical properties under a applied magnetic field, e.g, the half-quantum vortices^7^, Majorana zero-energy modes^8^, Skyrmionic states^9, 10^ and coreless vortices^11^, which is analog to the Anderson-Thouless vortices^12^ and Mermin-Ho vortices^13^ in the A phase of liquid ^3^He.

In addition, possible existences of odd-frequency spin-triplet even parity (OTE) states in the chiral p-wave superconductor *Sr*_2_ *RuO*_4_, e.g., the odd-frequency spin-triplet s-wave pairings at a vortex core has been discussed by several research groups^14–18^. It is known that superconducting pairing amplitude has to be an odd function with respect to the permutations of electrons spin and position for equal times, as required by Pauli’s exclusion principle. In an inhomogeneous system, translational invariance was broken-down, which would lead to couplings between the even- and odd-parity pairing states. With the spin rotation symmetry invariant, the pair amplitude of opposite parity must be opposite in the Matsubara frequency that defines the frequency of the relative motion of two electrons forming a Cooper pair^19^. A spin-singlet even-parity and a spin-triplet odd parity pairing correlation that is even in Matsubara frequency obeys that rule, and so is the odd-frequency spin-triplet s-wave pairing state.

In the chiral p-wave superconductor the structure of quasiparticle around a vortex core is closely associated with the bulk topology of the pairing states and the vortex topology. Indeed, it has been showed that an odd-frequency triplet s-wave core state has an identical spatial distribution as that of the local density of states at both zero-energy and finite energy levels for a single vortex winding antiparallel to the chirality of the *p*_+_ state in an atomic length scale, whereas it is not the situation for the occupied states for the vortex winding parallel to the *p*_+_ chirality^18^. Hence, on one side, one believes that the results^18^ strongly support for the existence of an odd-frequency spin-triplet s-wave core state in *Sr*_2_ *RuO*_4_ for an anti-winding vortex. On the other side, we should ask a question on what is the essence of the bound state at a parallel vortex core. Below a vortex with positive vorticity in relative to the chirality of *p*_+_ -state is defined as a parallel vortex, and a vortex with negative vorticity is known as an anti-parallel one.

Noticing also that almost all of the odd-frequency spin-triplet s-wave state and the Majorana zero-energy excitations are necessarily to be bound with an external topology defect (a vortex core, for instance) where the p-wave pairing amplitudes vanish or possess a local minimum. A second question arisen is if one can create an OTE bound state without involving local external topology defects in superconductor *Sr*_2_ *RuO*_4_. Alternatively, one may ask whether the odd-frequency spin-triplet s-wave state and the Majorana zero-energy excitation can exist at a distance from the vortex core. For this end, we use the Bogoliubov-de Gennes(BdG) model^20^ to solve the problem of a cylindrical chiral p-wave superconductor with a two-dimensional isotropic Fermi surface in the presence of a single vortex. We show that an odd-frequency spin-triplet s-wave core state exists for an anti-parallel vortex, in agreement with those reported in previous investigations^18^. However, we found out that the state at the core is dominated by the odd frequency triplet d-wave for a parallel vortex. We also investigate the effect of a local non-magnetic potential on the bound states. Remarkably, a zero-energy peak in the local density of states appears at a distance from the vortex core and from the local potential well. We found that the spatial distribution of the corresponding zero-energy local density of states(LDOS) peak fits well with that of a mixed odd-frequency spin-triplet s-wave and even-frequency p-wave amplitude. To our knowledge, this is the first zero-energy OTE pairing state coexisting with the bulk *p*_*x*_ ± *ip*_*y*_ pairing amplitudes in the chiral p-wave superconductor *Sr*_2_ *RuO*_4_.

The paper is organized as follows. The mixed s- and d-wave state with odd-frequency and spin-triplet symmetry is induced at the vortex core in chiral p-wave superconductive disks and this bound state can be manipulated by introducing a local non-magnetic potential barrier are discussed in Sec. Numerical Results. Our results are summarized in Sec. Conclusion. Finally, in Sec. Theoretical Approach, we present in detail our theoretical approach and model.

## Numerical Results

We discuss first the properties of bound states around an axial vortex. For an antiparallel vortex with *ν* = −1 we obtain the results (not shown) that are in good agreement with those in ref. 17. One of the interesting results is that an odd-frequency triplet s-wave amplitude has been induced at the vortex core. The OTE s-wave core state has an identical spatial profile as that of the local density of states at zero-energy and at finite energies even in an atomic length scale, which is believed as one of the evidences for the odd-frequency triplet s-wave pairings at the vortex core.

**Figure 1:**
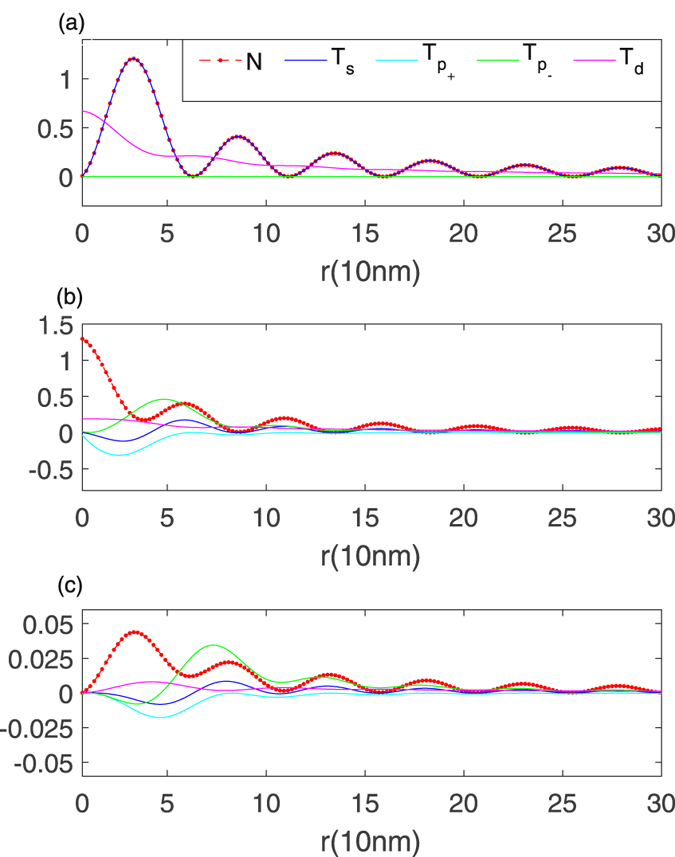
Spatial dependence of LDOS *N*(*r*, *E*) (red dot and dash lines), *T*_*s*_(*r*, *E*) (blue lines), ${T}_{{p}_{\pm }}(r,E)$ (cyan lines and green lines) and *T*_*d*_(*r*, *E*) (pink lines) for the zero-energy bound state (**a**), the first (**b**) and second (**c**) occupied states, respectively. A single vortex with vorticity *ν* = 1 is applied to a chiral p-wave superconductive disk with radius R.

While the identical spatial profile between the odd-frequency triplet s-wave amplitude and the LDOS remains for the zero-energy mode, it disappears at finite energy levels for a parallel vortex with *ν* = 1 (Fig. 1). It is further noticed that the LDOS peak is dominated by the first occupied state, instead of the zero energy mode. Therefore, different OTE states are required to understand those phenomena. As shown in Fig. 1, among possible pairing states concerned, only the odd-frequency d-wave pairing amplitude has a peak at the core center. These results lead us to speculate that the core state should be a mixed d-wave and s-wave state with the odd-frequency and spin-triplet symmetry. We present plausible arguments below.

In the presence of a vortex with vorticity *ν* a general expression for the pair potential is given by ${\rm{\Delta }}={{\rm{\Delta }}}_{0}{\rm{\Phi }}(\theta )F(r){e}^{(i{l}_{z}\theta )}{e}^{(iv\varphi )}$ for the chiral superconductor with an intrinsic angular momentum *l*_*z*_. It has been shown that the total angular momentum tends to *l*_*z*_ + *ν* at the vortex core center^21^. In the chiral p-wave superconductor *Sr*_2_ *RuO*_4_ under investigated, the degenerate states *p*_*x*_ ± *ip*_*y*_ with opposite chirality of *l*_*z*_ = 1 and *l*_*z*_ = −1 coexist. Therefore, the core states with angular momentum of 2 and 0 are expected for a single vortex with *ν* = 1. It is also noticed a single vortex in a superconductor can be regarded as a hole in the bulk. State parity conversion between the even and the odd occurs with the aid of Andreev reflections at the hole-superconductor boundary (surface). Therefore, states with even parity can exist around the vortex core. Importantly, the even parity triplet pairing state must be an odd function in frequency to satisfy Pauli’s exclusion principle. Indeed, as shown in our calculations, the zero energy mode is an OTE s-wave pairing state, and the first occupied state is dominated by the odd-frequency triplet d-wave state.

We now discuss effects of a non-magnetic potential on the spectrum and bound state properties of the system. We focus to the case of a parallel vortex with *ν* = 1. To manipulate the odd-frequency triplet core states, we increase steadily the potential magnitude until to the gap closing occurs(The parameters are $\gamma =\frac{R}{2}$, $\beta =\frac{1}{2}$ and *P*_*max*_ = 1.469 *eV*). Here, the term “gap closing” refers to all of the pairing amplitudes we concerned vanish at the core center. We choose a non-magnetic potential with Gaussian distribution to study how a local potential affect the topology of the bound state. The non-magnetic potential changes the translation invariance properties of the system. As a result, pairing states with even- and odd-parity would coexist. This is one of the mechanisms for the existence of a triplet bound state with even-parity. We find a relative wide range of parameters (the location and amplitude of the potential) can work, provided that the radius of the non-magnetic potential of ring-shaped is neither too small nor too large, e.g. in the range of $(\frac{R}{4} < \gamma  < \frac{3R}{5})$. Within this range, there is an approximately linear relationship between the magnitude and the radius of the potential for gap closing occurrence. Furthermore, one way to make a ring-shaped potential is to use a standard STM tip scanning with the desired radius over the *Sr*_2_ *Ru*_4_ disk, which gives a ring-shaped potential with height/amplitude of about electron-volts. Another way is to irradiate the sample with a non-paraxial x-ray with a desirable spot-size.

**Figure 2:**
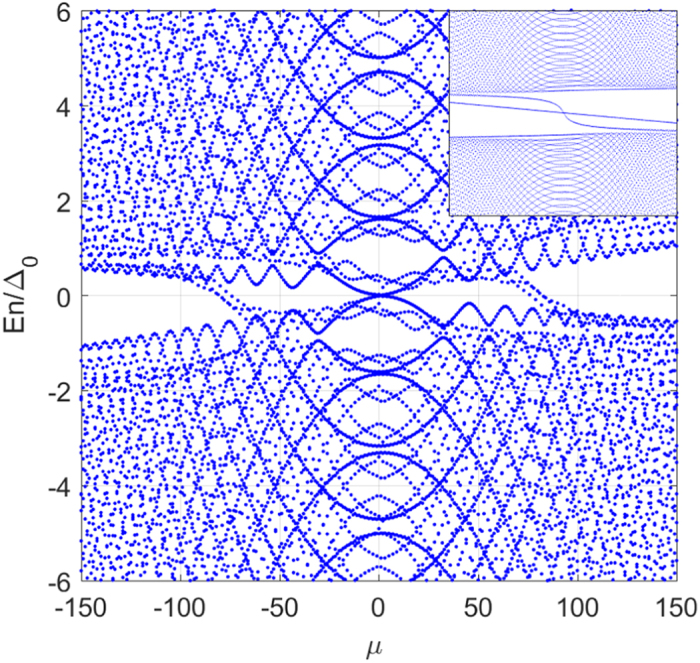
The quasiparticle excitation spectrum *E*_*n*_ as a function of the angular momentum under a non-magnetic potential well at *r* = *R*/2 and a vortex with vorticity *ν* = 1 at *r* = 0. The inset is the spectrum in the absence of the barrier.

Figure 2 shows the corresponding quasi-particle spectrum. The inset is the spectrum in the absence of the local potential. Clearly, an oscillating sub-gap “edge state” appears. This is reflected as a resonance in the LDOS spectrum (see below). Besides, two more pairs of zero energy modes have been induced because of the non-magnetic potential. Since we cannot distinguish an empty state from an occupied state with zero energy, the topological index (or the Chern number) that is associated with the geometric phase obtained by wave function of the occupied state along a closed loop in the phase space may become ill-defined. It infers that the bulk topological number may change discontinuously, depending on the topology of the Fermi surface. When it happens there always exists the zero-energy “edge state” with distinctive properties^22^.

**Figure 3:**
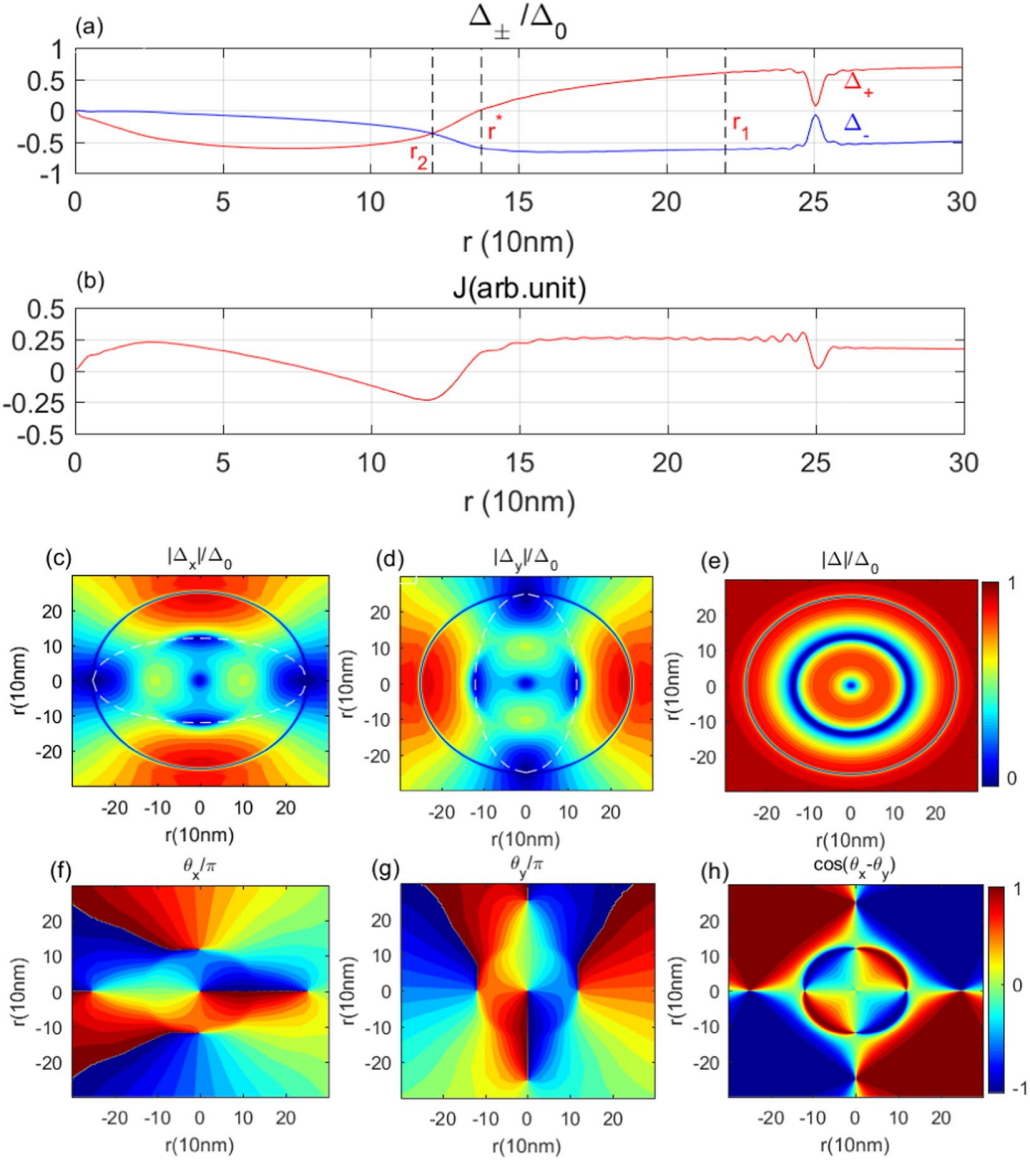
Profiles of p-wave potential and the microscopic current density. (**a**) Radial length dependence of the pair potential amplitude Δ_+_(*r*) and Δ_−_(*r*) under a single vortex with vorticity *ν* = 1 and a non-magnetic barrier at *R*/2. (**b**) Current distributions as a function of the radial length. (**c**,**d**,**e**) Contour plots for the amplitude of Δ_*x*_, Δ_*y*_, and Δ, respectively. (**f**,**g**) Spatial distributions of the phases in Δ_*x*_ and Δ_*y*_. (**h**) Profile of the phase difference in Δ_*x*_ and Δ_*y*_.

As aforementioned, a formal expression for the pairing potential of the *p*_*x*_ ± *ip*_*y*_ state is ${{\rm{\Delta }}}_{+}={{\rm{\Delta }}}_{+}(r){e}^{i\varphi }$ and ${{\rm{\Delta }}}_{-}(r){e}^{i3\varphi }$ when an axial vortex with *ν* = 1 is applied to the superconductor sample. Here, Δ_+_(*r*) and Δ_−_(*r*) are both real functions, which are evaluated from the simulations. The calculated Δ_+_(*r*) and Δ_−_(*r*) are shown in Fig. 3(a). Denoting Δ_±_ as Δ_*x*_ ± *i*Δ_*y*_. It is straightforward for us to show that Δ_*x*_ has, besides the one at the origin (0, *ϕ*) of the polar coordinate, four zeros at ((*r*_1_, 0), (*r*_1_, *π*)) and at ((*r*_2_, *π*/2)), ((*r*_2_, 3*π*/2)) while Δ_*y*_ has zeroes at ((*r*_2_, 0)), ((*r*_2_, *π*)) and at ((*r*_1_, *π*/2)), ((*r*_1_, 3*π*/2)). Where *r*_1_ is a radial length at which ${{\rm{\Delta }}}_{+}(r)=-{{\rm{\Delta }}}_{-}(r)$, whereas ${{\rm{\Delta }}}_{+}(r)={{\rm{\Delta }}}_{-}(r)$ at *r*_2_. Contour plots for |Δ_*x*_| and |Δ_*y*_| are shown in Fig. 3(c) and (d). Since both Δ_−_ and Δ_+_ are differentiable there must exists a closed loop *L*_1_ (*L*_2_) (white dashed lines) where the amplitude of Δ_−_(Δ_+_) approaches to zero-value asymptotically. The intersections in *L*_1_ and *L*_2_ are the so-called “singularities” where the relative phase *θ*_*x*_ − *θ*_*y*_ in the phase of Δ_*x*_ and Δ_*y*_ changes abruptly and discontinuously.

**Figure 4:**
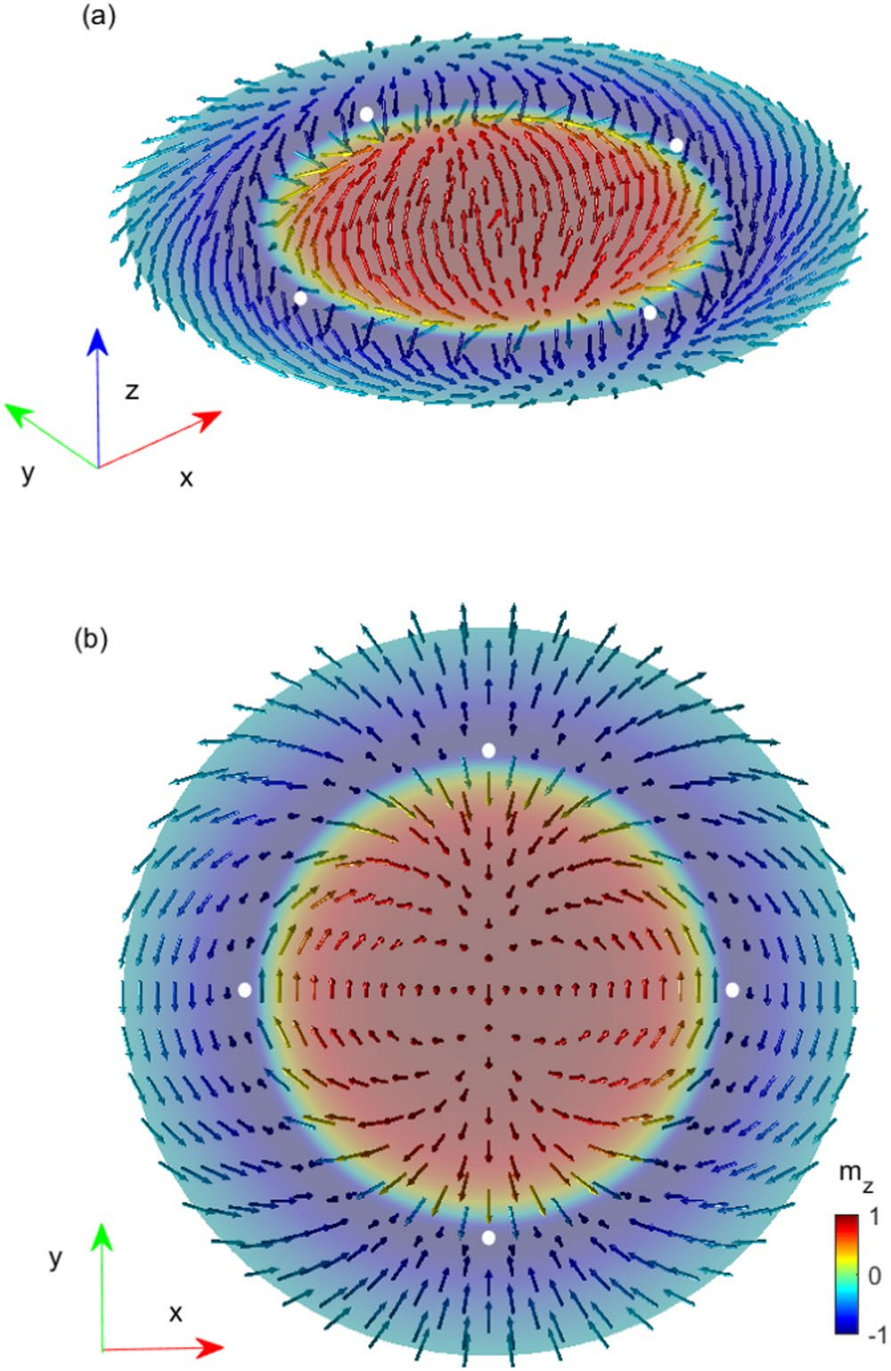
Spin-field profile of the skyrmion mode in the range (0, 0.4 R). The pseudo-spin vector ${\bf{m}}({\bf{r}})={{\boldsymbol{\Delta }}}^{\dagger }\overrightarrow{\sigma }{\boldsymbol{\Delta }}/{{\boldsymbol{\Delta }}}^{\dagger }{\boldsymbol{\Delta }}$ calculated using Δ_±_ with $\overrightarrow{\sigma }$ the pauli matrices. The colors show the amplitude of the z-component of **m(r)**. (**a**) 3D image. (**b**) Top view of the 3D image. White dots indicate the position of skyrmion modes.

**Figure 5:**
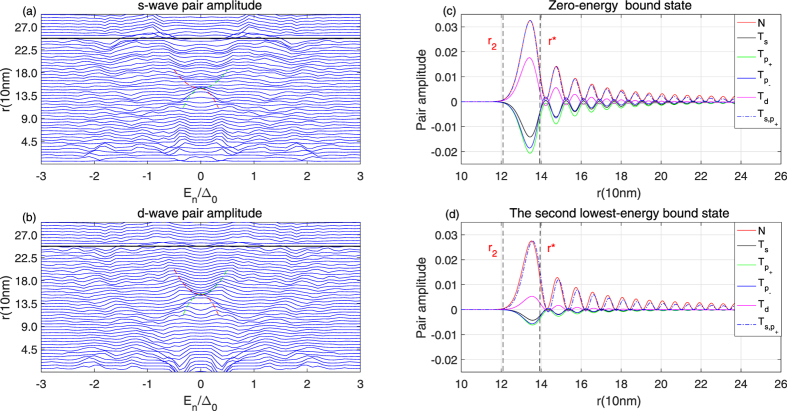
Spatial distributions for the local electron density and pairing amplitudes concerned are examined in an atomic length scale. (**a,b**) LDOS spectra for the odd-frequency triplet s- and d-wave pairings induced in a chiral p-wave superconductive disk under a single vortex with vorticity *ν* = 1 at *r* = 0 and a non-magnetic potential barrier at *R*/2. (**c,d**) Spatial dependence of LDOS *N*(*r*, E) (red lines), triplet s-wave amplitude *T*_*s*_(*r*, *E*) (black lines), p-wave ${T}_{{p}_{\pm }}(r,E)$(green lines and blue lines), triplet d-wave amplitude *T*_*d*_(*r*, *E*) (pink lines) and amplitude of mixed s-wave and *p*_+_-wave(blue dot and dash lines) for the zero-energy bound state (**c**) and the second lowest-energy bound state (**d**), respectively.

Spatially separated zeroes in Δ_*x*_ and Δ_*y*_ lead us to think of topological defects, which opens possibilities for the induced vortex with distinctive topological index. As well-known, the relative phase *θ*_*x*_ − *θ*_*y*_ must be an integer multiple of 2*π* radians at an Abrikosov vortex core. For instance, as shown in Fig. 3(f) and (g), *θ*_*x*_ and *θ*_*y*_ change 2*π* radians at the core of a single vortex with ν = 1. Meanwhile, |Δ_*x*_| and |Δ_*y*_| vanish simultaneously at the vortex core. We checked our data carefully and confirmed that *θ*_*x*_(*θ*_*y*_) could be a *π* winding at the half-vortex core. For instance, as shown in Fig. 3(f) and (g), *θ*_*y*_ is a 2*π* winding while *θ*_*x*_ is a *π* wingding at (*r*_2_, 0) and at (*r*_2_, *π*). Also *θ*_*x*_ is a 2*π* winding while *θ*_*y*_ is a *π* wingding at (*r*_1_, *π*/2) and at (*r*_1_, 3*π*/2). Therefore, the resulting *θ*_*x*_ − *θ*_*y*_ is a *π* winding there. In contrast to it, *θ*_*x*_ − *θ*_*y*_ changes only *π* radians [Fig. 3(h)] and only one of the Δ_*x*_ and Δ_*y*_ amplitudes decays to zero [Fig. 3(c) and (d)] at the new topological vortex core, which imply that the induced vortex can be classified as to a half-quantum ($\frac{1}{2}$) vortex. We would further point out that the $\frac{1}{2}$-vortices can be distinguished according to theirs winding direction with respective to the chirality of the pairing states. While the half-quantum vortex winding parallel to the chirality of bulk *p*_+_ pairing states is knownn as a $\frac{1}{2}$ -vortex, the one winding antiparallel to the chirality is an $\frac{1}{2}$-antivortex. Importantly, the $\frac{1}{2}$-vortex and $\frac{1}{2}$-antivortex always appear in pairs [see, Fig. 3(f) and (g)], thereby guaranteeing winding number conservation. More surprisingly, the half vortex distributes uniformly on a circle with a radius of *r*_1_, while the half antivortex is on a circle with a radius of *r*_2_, forming the so-called skyrmion modes (see Fig. 4 below). By using the expression for the topological charge of skyrmions^23^, we find an integer topological charge *Q* = 2 for the skyrmions associated with the $\frac{1}{2}$-vortex, whereas *Q* = −2 for the $\frac{1}{2}$-antivortex skyrmions. To further show the skyrmion structure, we present the spatial distribution of the current calculated self-consistently in Fig. 3(b) and the so-called spin-field profile of the skyrmion (Fig. 4(a,b)). Clearly, the current changes sign and the spin-field reversing occurs as the electron local coordinate goes across the domain-wall at r*. Interestingly, the skyrmion modes are analog to the dynamical skyrmions discussed by Giordano *et al*.^24^. The dynamical skyrmions treated as non-linear gyrotropic rotations of the magnetic vortex core that give rise to spin-waves with spiral spatial profile. The spin-field aligning or the *θ* spirals two complete cycles around the domain wall C(r*) in our static skyrmion mode, which would have a resonance spectrum in the local density of states (see, Fig. 5).

Let us examine *ϕ*-dependence of the relative phase difference around the cycle *C*(r*) where the amplitude of Δ_+_ tends to zero. It indicates that *θ*_*x*_ − *θ*_*y*_ is a constant of $\frac{\pi }{2}$ on *C*(r*) [Fig. 3(h)]. *θ*_*x*_ − *θ*_*y*_ varies from 180 degrees to 0 degrees when electron local coordinates increase crossing through the cycle *C*(r*) in the second and fourth quadrants. Instead, it varies from 0 degrees to 180 degrees in the first and third quadrants. It increases that phases of Δ_*x*_ and Δ_*y*_ precede $\frac{\pi }{2}$ radians alternately in adjacent quadrants of the polar coordinates. Therefore, an incident chiral *p*_−_ state will be reflected on the circle to its mirror state with an opposite chirality. Superposition of the incident with its mirror particles leads to transverse resonances in the LDOS spectra, as shown in the left panel of Fig. 5. Red and green lines highlight the first resonant peak. They intersect at zero-energy, resulting in a new zero-energy DOS peak at a distance away from the external vortex center and away from the potential-well location.

We discuss the major physical processes that lead to resonances in the spectra briefly by examining electrons scattered on *C*(r*). Sandwiched between two superconductive domains, the cycle *C*(r*) where bulk *p*_+_ pairing amplitude vanishes may be regarded as an electronic subsystem of”normal state” domain wall. Electrons scattered on the wall have suffered to two processes. One is the normal reflection. Another is the Andreev retroflection. At the temperature much lower than the superconducting transition temperature the Andreev reflection dominates. In fact, it is the Andreev reflection that assists the bound state carrying the local microscopic current screening the magnetic field of the spontaneous half-quantum vortices. Consider an incident electron with an angle *α* measured from the polar axis. In general, the total current carried by the bound state can be expressed as ${{\rm{\Sigma }}}_{n,\alpha }{I}_{n}(\alpha )\sin (n\theta )$ with *n* being an integer. Since each bound state with (*α*, *θ*) has a mirror state of $(-\alpha ,\theta \pm \pi )$, the odd harmonics of the normal direction current $({j}_{\perp })$ will cancel each other. To the leading order, it is proportional to $\sim {I}_{2}(\alpha )\sin \,(2\theta )$. It is therefore seen that the state $\frac{1}{\sqrt{2}}|{{\rm{\Delta }}}_{x}\pm i{{\rm{\Delta }}}_{y}\rangle$ is a stable bound state with ${j}_{\perp }=0$ at $\theta =\pm \pi /2$, leaving a transverse current ${j}_{\phi }\sim {I}_{2}(\alpha )\sin (\alpha )\sin \,(2\theta )$ that oscillates two complete circles/periods when electron local coordinates spiral one turn around the *C*(r*). Spatially inhomogeneous current distribution implies a time-dependent local electron density, according to the current continuality relationship. A resonant LDOS spectrum is obtained once the Fourier transformation has been performed with respect to the time variable.

To identify the bound state that is responsible for the new DOS peak, spatial distributions for the local electron density and pairing amplitudes concerned are examined in an atomic length scale. As shown in the right panel of Fig. 5, both the zero-energy odd-frequency triplet s- and d-wave amplitudes have been significantly enhanced, in comparison with those in the absence of the local potential barrier. A more close examining reveals that among various pairing amplitudes concerned only the sign of the triplet s-wave amplitude and the chiral *p*_+_-wave amplitude remains unchanged over the whole length scale. Hence, an identical r-dependence of the local electron density with that of a mixed s- and p-wave triplet amplitude (${T}_{s,{p}_{+}}=0.36{T}_{s}+0.64{T}_{{p}_{+}}$) is expected in an atomic length scale when the mixed pairing amplitude is normalized by the maximum electron density at different quantized energy levels of the bound states. Therefore, we propose that the mixed state of odd frequency triplet s-wave and the even-frequency chiral p-wave is responsible for the local DOS peak at both zero-energy and finite energy levels.

It is important to further note that the particle-hole symmetry of the system Hamiltonian remains hold even in the presence of an non-magnetic potential. By using this property, it is readily for us to prove that the Majorana condition ${u}_{n}(r)={v}_{n}^{\ast }(r)$ is satisfied at zero-energy provided that there exists no pairing states breaking down the time-reversal in-variance locally. We compared *u*_*n*_(*r*) with ${v}_{n}^{\ast }(r)$ in the zero-energy state. Indeed, there exists some derivations between them in the atomic length scale. It means, strictly speaking, Majorana condition is not satisfied if a mixed triplet s- and p-wave state is responsible for the zero-energy mode. However, possible existence of the Majorana mode remains expected if the p-wave component could be suppressed by embedding a non-magnetic quantum dot on the cycle where the zero-energy peak locates. We would point out that the Majorana condition is likely to be recovered after taking the points below into consideration. Firstly, the $\frac{1}{2}$-vortex and $\frac{1}{2}$-antivortex always exist in pair in present case. Therefore, existences of the $\frac{1}{2}$-vortex and $\frac{1}{2}$-antivortex pairs don’t break down the winding number conservation. Secondly, we can embed some non-magnetic impurities (quantum dots) uniformly on the C(r*) to suppress the time-reversal violation state of the chiral *p*-wave amplitude. Very importantly, while the p-wave amplitude would be greatly suppressed with an increase of the scattering intensity of the non-magnetic impurities, the zero-energy odd-frequency triplet s-wave pairing amplitude that is insensitive to the scattering intensity remains unchanged. Then, using a TEM probe of s-wave superconductor the zero-energy odd-frequency triplet s-wave state can be tested as a zero-bias LDOS peak. When this happens the zero-energy LDOS signaled is a good indication of the Majorana excitation without an external magnetic topology defect.

## Conclusion

We have investigated the properties of bound states in chiral p-wave superconductive disks. An odd-frequency triplet even parity (s- and/or d-wave) bound state or the zero-energy Majorana fermion is found at a single Abrikosov-type vortex core. The zero-energy OTE can exist at a distance from the external topological defect when a non-magnetic potential with appropriate amplitude has been included in the model Hamiltonian. Remarkably, half-quantum antivortex-vortex pairs and skyrmion modes are found to exist with the aid of the Andreev reflections. The topological charges have equal magnitudes but opposite signs for the $\frac{1}{2}$-antivortex and $\frac{1}{2}$-vortex skyrmion modes, satisfying winding number conservation law, as expected.

## Theoretical Approach

We begin with the BdG equations^20, 25, 26^ for the chiral p-wave superconductor *Sr*_2_ *RuO*_4_ with pairing symmetry of *p*_*x*_ ± *ip*_*y*_:

1a $${H}_{e}{u}_{n}-\frac{i}{{k}_{F}}\sum _{\pm }\,[{{\rm{\Delta }}}_{\pm }{\square}_{\pm }+\frac{1}{2}({\square}_{\pm }{{\rm{\Delta }}}_{\pm })]{v}_{n}={E}_{n}{u}_{n},$$

1b $$-{H}_{e}^{\ast }{v}_{n}-\frac{i}{{k}_{F}}\sum _{\pm }\,{[{{\rm{\Delta }}}_{\pm }{\square}_{\pm }+\frac{1}{2}({\square}_{\pm }{{\rm{\Delta }}}_{\pm })]}^{\ast }{u}_{n}={E}_{n}{v}_{n}.$$

Where the single particle Hamiltonian is ${H}_{0}=\frac{1}{2m}{(-i{\rm{\nabla }}+\frac{e}{c}{\bf{A}})}^{2}-e{{\bf{A}}}_{0}-\mu$, *m* is the electron mass, *e* is the electron charge, and *c* is the speed of light in vacuum *μ* is the chemical potential. **A** is the vector potentials. The gap equation of *p*_*x*_ ± *ip*_*y*_-wave is written as^20^

2 $${{\rm{\Delta }}}_{\pm }({\bf{r}})=-i\frac{gS}{2{k}_{F}}\sum _{{E}_{n} < \hslash {\omega }_{D}}[{v}_{n}^{\ast }({\bf{r}}){\square}_{\mp }{u}_{n}({\bf{r}})-{u}_{n}({\bf{r}}){\square}_{\mp }{v}_{n}^{\ast }({\bf{r}})]\times [1-2f({E}_{n})].$$

Δ_±_ is the order parameter for the *p*_*x*_ ± *ip*_*y*_-wave, *g* is the coupling constant, ${k}_{F}=\sqrt{2m{E}_{F}/{\hslash }^{2}}$ is the Fermi wave length, ${\square}_{\pm }={e}^{\pm i\phi }(\frac{{\rm{\partial }}}{{\rm{\partial }}r}\pm \frac{i}{r}\frac{{\rm{\partial }}}{{\rm{\partial }}\phi })$, ${E}_{n}$ is the energy eigenvalue for the superconducting quasiparticle, and $f({E}_{n})=[1+{e}^{{E}_{n}/{k}_{B}T}]-1$ is the Fermi distribution function. The summations in equation are over all the quasiparticle states with energies in the Debye window. For simplicity, we assume $\hslash =1$, ${k}_{B}=1$ and the superconductor is two dimensional which has a cylindrical Fermi surface. The vector potentials obey the Maxwell equation:

3 $${\rm{\nabla }}\times {\rm{\nabla }}\times {\bf{A}}({\bf{r}})=\frac{4\pi }{c}{\bf{J}}({\bf{r}}).$$

Here,we use the gauge ${\rm{\nabla }}\cdot {\bf{A}}\,=\,0$. The supercurrent density is calculated by

4 $${\bf{J}}({\bf{r}})=\frac{e\hslash }{2mi}\sum _{n}\{f({E}_{n}){u}_{n}^{\ast }({\bf{r}})[{\rm{\nabla }}-\frac{ie}{\hslash c}{\bf{A}}({\bf{r}})]{u}_{n}({\bf{r}})+(1-f({E}_{n})){v}_{n}({\bf{r}})[{\rm{\nabla }}-\frac{ie}{\hslash c}{\bf{A}}({\bf{r}})]{v}_{n}^{\ast }-h.c.\}.$$

We consider the problem of a chiral p-wave superconductor disk of radius **R** under an axial vortex of vorticity ν. In the cylindrical coordinate electron and hole quasi-particle wave functions ${u}_{n}({\bf{r}})$ and ${v}_{n}({\bf{r}})$ can be expanded in terms of Bessel function basis.

5a $${u}_{n}({\bf{r}})=\sum _{j}{a}_{n\mu j}{\varphi }_{j\mu }(r){e}^{i\mu \varphi },$$

5b $${v}_{n}({\bf{r}})=\sum _{j}{b}_{n{\mu }^{{\rm{^{\prime} }}}j}{\varphi }_{j{\mu }^{{\rm{^{\prime} }}}}(r){e}^{i{\mu }^{{\rm{^{\prime} }}}\varphi }.$$

Here ${a}_{n\mu j}$ and ${b}_{n\mu j}$ are expansion coefficients. ${\varphi }_{j\mu }(r)$ is given by ${\varphi }_{j\mu }(r)=\frac{\sqrt{2}}{R{J}_{\mu +1}({\alpha }_{j\mu )}}{J}_{\mu }\frac{r}{R}$, with ${J}_{\mu }$ is the *μ*_*th*_ Bessel function and ${\alpha }_{j\mu }$ is the ${j}_{th}$ zero of ${J}_{\mu }$. $\mu ,{\mu }^{{\rm{^{\prime} }}}\in Z$ are integers denoting the angular momentum quantum number of a constituting component of the quasi-particle function. Depending on the vorticity number, *μ* and *μ*′ have to satisfy the following relations in order to ensure a real gauge of the BdG model. For instance, *μ* = *μ*′ when *ν* = 1, $\mu ={\mu }^{{\rm{^{\prime} }}}+2$ when *ν* = −1, and $\mu ={\mu }^{{\rm{^{\prime} }}}+1$ when *ν* = 0 *etc*. Quasi-particle wave functions satisfy the normalization condition:

6 $$\int |{u}_{n}({\bf{r}}{)|}^{2}+|{v}_{n}({\bf{r}}{)|}^{2}d{\bf{r}}=1.$$

which put a constrain on the expansion coefficients ${a}_{n\mu j}$ and ${b}_{n\mu j}$. By using the particle-hole symmetry relation, electron densities of the BdG equation are as follows:

7 $$n({\bf{r}})=2\sum _{{E}_{n}}|{u}_{n}{({\bf{r}})}^{2}|\,f({E}_{n}).$$

From the self-consistent solutions, we can calculate the LDOS as

8 $$N({\bf{r}},E)=\sum _{n}[|{u}_{n}({\bf{r}}{)|}^{2}\delta (E-{E}_{n})+|{v}_{n}({\bf{r}}{)|}^{2}\delta (E+{E}_{n})].$$

Since an Abrikosov vortex breaks down the translation symmetry and leads to couplings between the even-parity and odd-parity pairing states, one expects then possibilities for an odd-frequency pairing bound state around the vortex even in a conventional spin-singlet s-wave superconductor. One of the manifestations of the bound state is the enhanced LDOS at the vortex core, as observed as a zero bias conductance peak in the scanning tunneling spectroscopy^27, 28^. With the same mechanism, the OTE bound states are allowable in a chiral p-wave superconductor. The odd-frequency spin-triplet s- and d-wave pair amplitudes are defined by ${T}_{s,triplet}=({T}_{s,\uparrow \downarrow }+{T}_{s,\downarrow \uparrow })/2$ and ${T}_{d,triplet}=({T}_{d,\uparrow \downarrow }+{T}_{d,\downarrow \uparrow })/2$ with

9a $${T}_{s,\uparrow \downarrow }({\bf{r}},E)=\sum _{n}{v}_{n}^{\ast }({\bf{r}}){u}_{n}({\bf{r}})\delta (E-{E}_{n}),$$

9b $${T}_{s,\downarrow \uparrow }({\bf{r}},E)=\sum _{n}{u}_{n}({\bf{r}}){v}_{n}^{\ast }({\bf{r}})\delta (E+{E}_{n}).$$

In analog to Eq.(2), one can formally write down the self-consistent condition for a d-wave pair potential^26^ from a standard BdG model, from which the odd-frequency spin-triplet d-wave pair amplitude is given by

10a $${T}_{d,\uparrow \downarrow }({\bf{r}},E)=-\frac{gS}{4{k}_{F}^{2}}\sum _{n}({v}_{n}^{\ast }({\bf{r}}){\rm{\Pi }}{u}_{n}({\bf{r}})+{u}_{n}({\bf{r}}){\rm{\Pi }}{v}_{n}^{\ast }({\bf{r}}))-2({{\rm{\partial }}}_{x}{v}_{n}^{\ast }({\bf{r}}){{\rm{\partial }}}_{x}{u}_{n}({\bf{r}})-{{\rm{\partial }}}_{y}{v}_{n}^{\ast }({\bf{r}}){{\rm{\partial }}}_{y}{u}_{n}({\bf{r}}))\delta (E-{E}_{n}),$$

10b $${T}_{d,\downarrow \uparrow }({\bf{r}},E)=-\frac{gS}{4{k}_{F}^{2}}\sum _{n}({u}_{n}({\bf{r}}){\rm{\Pi }}{v}_{n}^{\ast }({\bf{r}})+{v}_{n}^{\ast }({\bf{r}}){\rm{\Pi }}{u}_{n}({\bf{r}}))-2({{\rm{\partial }}}_{x}{u}_{n}({\bf{r}}){{\rm{\partial }}}_{x}{v}_{n}^{\ast }({\bf{r}})-{{\rm{\partial }}}_{y}{v}_{n}^{\ast }({\bf{r}}){{\rm{\partial }}}_{y}{u}_{n}({\bf{r}}))\delta (E+{E}_{n}).$$

where ${{\rm{\partial }}}_{x}=cos(\phi )\frac{{\rm{\partial }}}{{\rm{\partial }}r}-\frac{sin(\phi )}{r}\frac{{\rm{\partial }}}{{\rm{\partial }}\phi }$ and ${{\rm{\partial }}}_{y}=sin(\phi )\frac{{\rm{\partial }}}{{\rm{\partial }}r}+\frac{cos(\phi )}{r}\frac{{\rm{\partial }}}{{\rm{\partial }}\phi }$ in cylindrical coordinates. The operator ${\rm{\Pi }}$ is given by:

$${\rm{\Pi }}={{\rm{\partial }}}_{x}^{2}-{{\rm{\partial }}}_{y}^{2}=cos(2\phi )(\frac{{{\rm{\partial }}}^{2}}{{\rm{\partial }}{r}^{2}}-\frac{1}{r}\frac{{\rm{\partial }}}{{\rm{\partial }}r}-\frac{1}{{r}^{2}}\frac{{{\rm{\partial }}}^{2}}{{\rm{\partial }}{\phi }^{2}})+2sin(2\phi )(\frac{1}{{r}^{2}}\frac{{\rm{\partial }}}{{\rm{\partial }}\phi }-\frac{1}{r}\frac{{{\rm{\partial }}}^{2}}{{\rm{\partial }}r{\rm{\partial }}\phi }).$$

We note that the particle-hole symmetry relationship $\{{u}_{-{E}_{n}},{v}_{-{E}_{n}}\}=\{{v}_{{E}_{n}}^{\ast },{u}_{{E}_{n}}^{\ast }\}$ has been used in defining the triplet s- and d-wave pairs. Finally, a non-magnetic potential is introduced when necessary.

11 $$P(r,\gamma ,\beta )=\frac{1}{\beta \sqrt{2\pi }}{e}^{-\frac{{(r-\gamma )}^{2}}{2{\beta }^{2}}}.$$

This is the expression of normal distribution. $\gamma =R/2$ is the mean of the distribution and *β* is the standard deviation of the distribution. Different parameter values are used to adjust the location and potential intensity. The non-magnetic potential is extrmely located.

In our calculation, we set the coupling strength $g=0.115$, the cutoff energy ${E}_{c}={E}_{f}$. The coherence length ${\xi }_{0}$ is 66 *nm*, from which the coefficient in Eq. (3) is $\frac{4\pi m{\xi }_{0}^{4}}{{\hslash }^{2}{c}^{2}}=3.53\times {10}^{-6}$ with GL parameter $\kappa \equiv \frac{\lambda }{{\xi }_{0}}=2.372$. The calculation is performed for the sample of radius *R* = 500 *nm*.
